# Identifying circulating glioma cells and their clusters as diagnostic markers by a novel detection platform

**DOI:** 10.1002/ctm2.318

**Published:** 2021-02-04

**Authors:** Yangzhi Qi, Qian Sun, Gang Deng, Huikai Zhang, Yang Xu, Yuane Li, Shaoyi Huang, Yong Li, Zhang Ye, Yixuan Wang, Fanen Yuan, Ping Hu, Lun Gao, Hongxiang Jiang, Wenya Wu, Baohui Liu, Qianxue Chen

**Affiliations:** ^1^ Department of Neurosurgery Renmin Hospital of Wuhan University Wuhan Hubei P.R. China; ^2^ Key laboratory of Renmin hospital Wuhan University Wuhan Hubei P.R. China; ^3^ Department of Circulating Tumor Cells YZY Medical Technological Company Wuhan Hubei P.R. China

Dear editor:

Diffused glioma is the leading cause of central nervous system tumor‐related deaths worldwide.^1^ As a non‐invasive and convenient bio‐marker, despite that circulating tumor cells (CTC) and their clusters show great feasibility in tumor diagnosis and management,^2^ their isolation and identification remain challenging, limiting further clinical application.^3^, ^4^, ^5^, ^6^, ^7^ Therefore, more efficient detection platforms are urgently required in this field. This inspired us to fabricate a novel platform to detect circulating glioma cells and their clusters for further evaluated their value in tumor diagnosis and management, and highlight the wider potential of CTC for clinical application.

In this study, we performed a novel isolation method by size of epithelial tumor cells device. The isolation is carried out using a biocompatible parylene polymer membrane with a pore diameter of 8 μm under a high flow rate, enriching for CTCs without requiring tumor cell‐specific capture antibodies.

To test the capture efficiency of this device, two common malignant glioma cell lines, U87 and U251, were selected and spiked into healthy donors’ blood samples. In detecting spiked U87 cells from blood samples at concentrations of 5, 10, 20, 50, 100, and 150 cells per 5 ml, the capture efficiency were 86.0%, 87.0%, 86.5%, 86.0%, 91.3%, and 92.9%, respectively. The capture efficiency were 80.0%, 77.0%, 81.5%, 78.6%, 84.3%, and 86.2% when detecting U251 cells from blood samples at above concentrations (Figure 1A‐C). Besides, CTC cluster was not observed in any test, confirming that it was not artifact.

**FIGURE 1:**
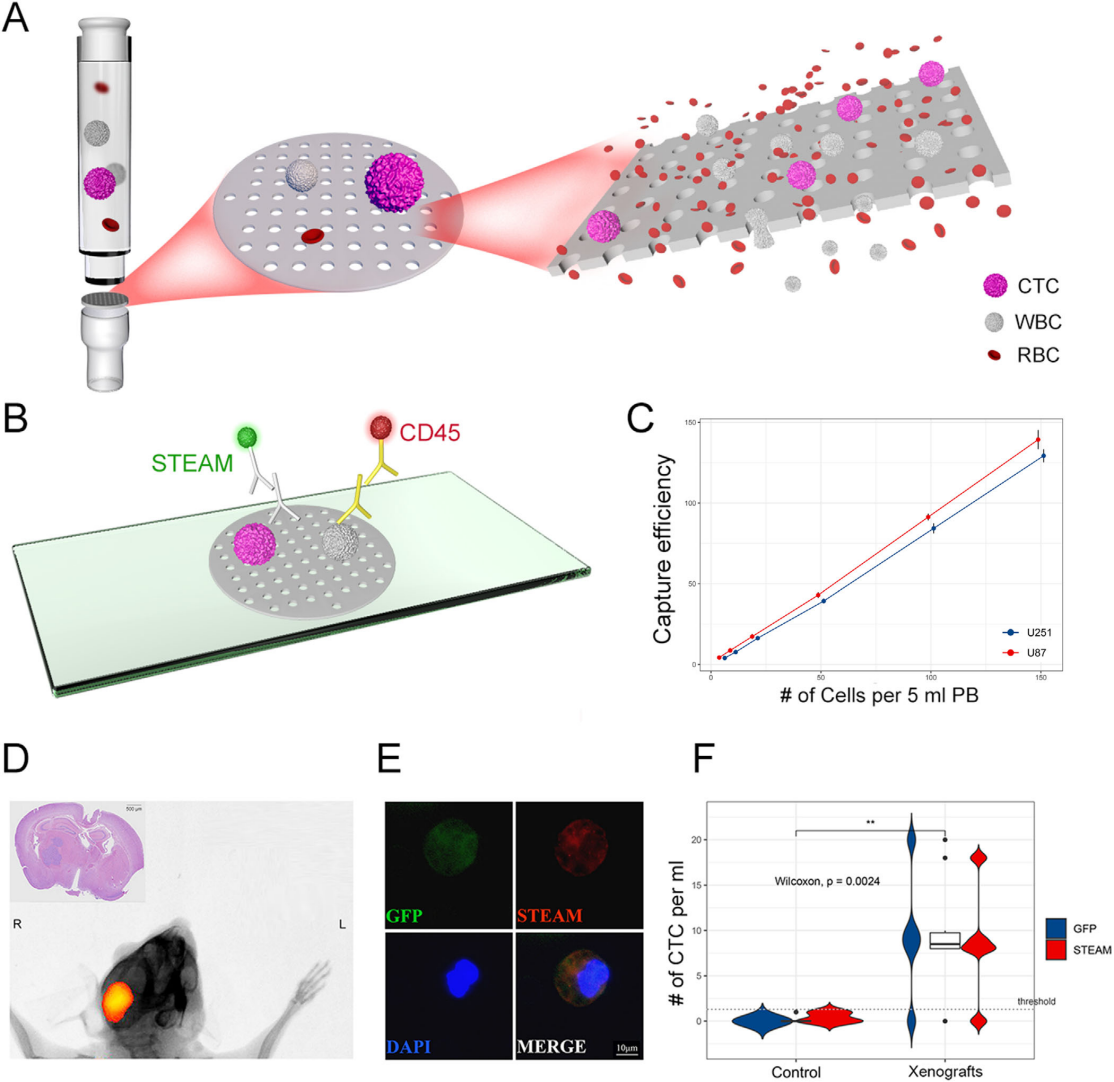
Operational principle of the platform, in vitro, and in vivo experiments. A, The isolation is carried out using a biocompatible parylene polymer membrane. B, Immunofluorescence staining for identification of CTC and WBC. C, In vitro test: for the U87 cell line, the capture efficiency was 86.0%, 87.0%, 86.5%, 86.0%, 91.3%, and 92.9%, respectively. For the U251 cell line, the capture efficiency was 80.0%, 77.0%, 81.5%, 78.6%, 84.3%, and 86.2%, respectively. Each test was repeated three times. D, In vivo test: a representative fluorescence in vivo imaging of a mouse xenograft and HE staining of its brain. E, Representative image of mouse derived STEAM+/DAPI+/CD14,16,45‐ CTC. Scale bar = 10 μm. F, Quantification of STEAM+ cells and GFP+ cells in blood samples from sham‐injected mice (n = 5) and xenografts (n = 5). Threshold: 2 STEAM+ CTC per mL PB

We then investigate feasible identification methods for glioma CTC. Our data showed that Wright's staining, one convincing and feasible identification method,^8^ could not distinguish glioma CTC from other brain tumors’, indicating that identification methods based on tumor features cannot define CTC origin, which limit its diagnostic value (Figure 4A‐C; Tables S1 and S2). However, highly specific identification methods targeted GBM‐derived CTC, represented by STEAM staining, an antibody cocktail based on differential expressed genes between CTC and WBC, would increase the risk of false positives and high background levels due to healthy cells expressing several of the included markers.^3^ Therefore, we combined STEAM staining with a series of criteria of malignant features for CTC,^8^ and subsequently proved that modified STEAM staining can remarkably decrease background levels in mouse experiments and healthy donors (Figure 1D‐F; Figure 2A,B; Figure 4D; Table S3), and was also more sensitive than Wright's staining (Figure 2C; Table S4).

**FIGURE 2:**
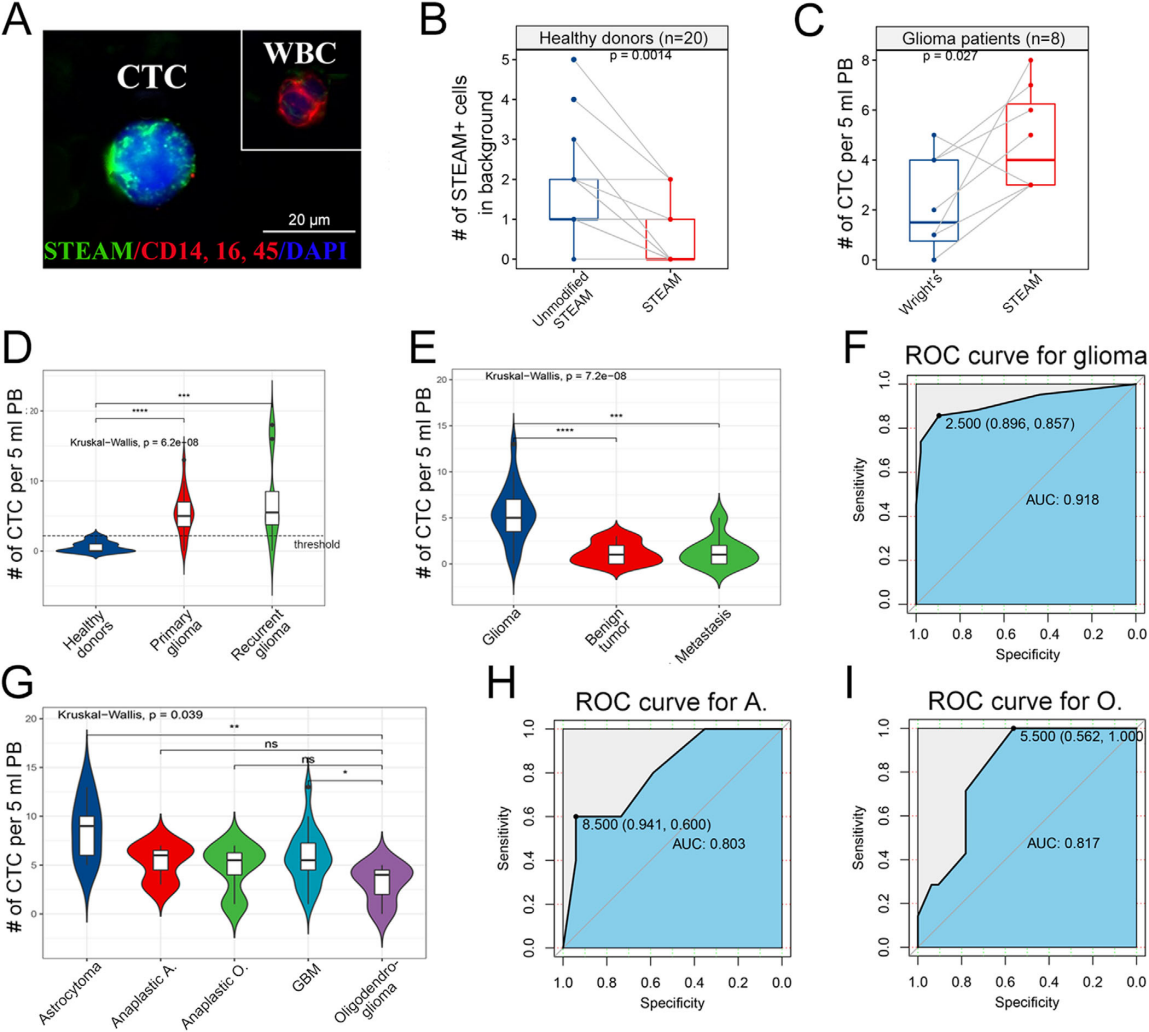
Detecting CTC by our platform in patients with glioma. A, Representative image of a STEAM+ CTC alongside a WBC isolated from a glioma patient's blood. Scale bar = 20 μm. B, Quantification of STEAM+ cells as background in healthy donor (n = 20) by unmodified STEAM staining and improved STEAM staining (*P* < .01). C, Quantification of patients‐derived (n = 8) CTC in 5 ml PB by Wright's staning and improved STEAM staining respectively (*P* < .05). D, Quantification of STEAM+ cells in healthy donors (n = 20), primary glioma (n = 42), and recurrent glioma (n = 8) (*P* < .0001, *P* < .001, respectively). Threshold: 3 STEAM+ cells per 5 mL PB. E, Quantification of STEAM+ cells in patients with primary glioma (n = 42), benign brain tumor (meningioma = 12, schwannoma = 8), and metastasis (n = 8) (*P* > .05). F, ROC curve analysis reveals that STEAM+ CTCs have a high sensitivity and specificity to predict glioma (AUC = 0.918). G, Quantification of STEAM+ cells in 5 different pathologic subtypes of glioma. The level of STEAM+ cells in oligodendroglioma is significantly lower than that in astrocytoma and GBM (*P* < .01, *P* < .05, respectively). A: astrocytoma; O: oligodendroglioma. H, ROC curve analysis revealed that the level of STEAM+ cells can predict astrocytoma. (AUC = 0.803). I, ROC curve analysis revealed that the level of STEAM+ cells can also predict oligodendroglioma (AUC = 0.817)

We next examine the feasibility of our platform in glioma through clinical test. Of the 42 primary diffused glioma patients enrolled in this study, nearly 85.7% (36/42) had detectable CTC, remarkably higher than previous study (median: 5.0 cells per 5 ml, range: 0–13, mean: 5.5±3.0) (Figure 2D; Table S5). For patients with recurred glioma, 7/8 (87.5%) patients had detectable CTCs above threshold (median: 5.5 cells per 5 ml, range: 0–18, mean: 7.3±6.3) (Figure 2D; Table S3). To further investigate the feasibility of our platform in differential diagnosis, 20 patients with benign tumors and eight with brain metastasis were enrolled in this study. We found the level of STEAM‐positive CTC in the glioma was significantly higher than that in above‐mentioned brain tumors (*P* < .0001) (Figure 2E) and ROC curves revealed the value of CTC in predicting glioma CTC (Figure 2F). These findings demonstrated that STEAM‐positive CTC could be a highly specific diagnostic bio‐marker for diffused glioma.

Contrary to previous study, among five pathological subtypes, we found that CTC levels was higher in astrocytoma while lower in oligodendroglioma (Figure 2G‐I; Figure 4E).^4^, ^7^ This finding shed a new potential that using CTC predicting tumor pathological subtypes. After verified in a larger cohort, it could make the diagnosis more accurate.

We next sought to determine whether CTC cluster was an intrinsic property of glioma. Compared with the extremely low proportion of CTC‐CTC clusters,^9^ CTC‐WBC clusters occurred more frequently (Figure 3A; Figure 4F and 4G). This was the first report of a large number of CTC‐WBC clusters in glioma patients. Previous study reported that CTC can enable cell cycle progression by binding with neutrophils, highlighting CTC's ability to escape from anoikis and immune surveillance through making a stable niche with non‐tumor cells.^10^ We found that nearly 33.3% (14/42) of patients with primary glioma had CTC‐WBC clusters (median: 0 cluster per mL, range: 0–3, mean: 0.6±0.9) (Figure 3B, Table S3). In recurrent glioma, the proportion increased to 75% (6/8), with a median of 1 cluster per 5 ml (range: 0–2, mean: 0.9±0.6) (Figure 3B, Table S3), indicating that CTC‐WBC clusters could help monitoring recurrence. However, no difference was observed in different pathologic subtypes (Figure 3C). Subsequently, one blood sample was presented for Wright & Giemsa staining, showing that CTC associated WBCs were neutrophils, consistent with previous study (Figure 3D).^10^

**FIGURE 3:**
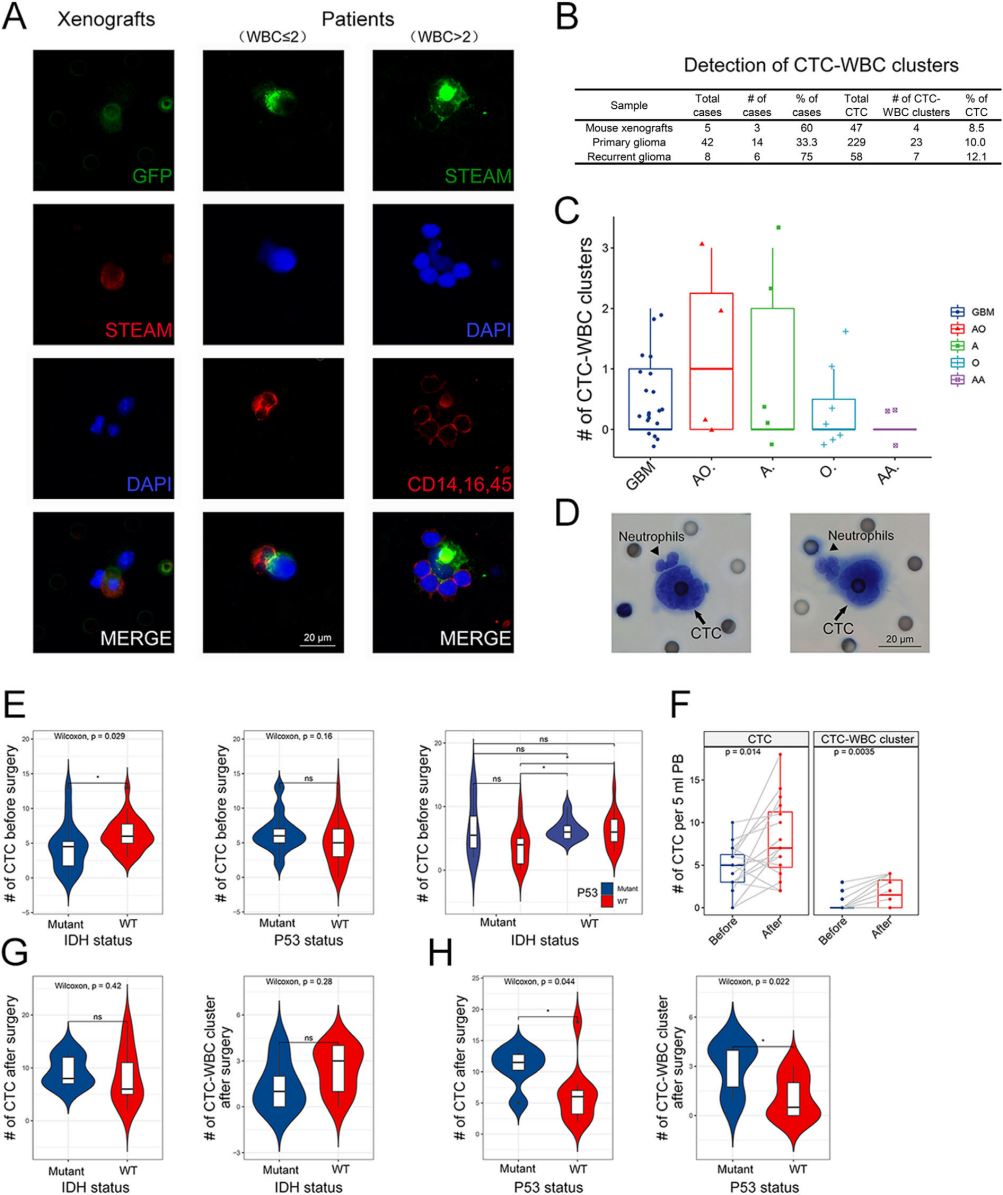
Investigating CTC‐WBC clusters and the correlation between clinical features. A, Left panel: representative image of CTC‐WBC clusters isolated from mouse xenografts. Middle panel: representative image of CTC‐WBC clusters isolated from glioma patients. The number of WBC ≤ 2. Right panel: representative image of CTC‐WBC clusters isolated from glioma patients. The number of WBC > 2. Scale bar = 20 μm. B, Quantification of detectable CTC clusters in xenografts (n = 5), primary glioma (n = 42), and recurrent glioma (n = 8). C, Quantification of CTC‐WBC clusters in five different pathologic subtypes of glioma. No difference was observed in the detectable level of CTC‐WBC clusters among each subtypes (*P* > .05). D, Representative image of CTC‐WBC clusters by Wright's staining showed that tumor associated WBCs were neutrophils. Scale bar = 20 μm. E, The level of CTC was related to IDH status (*P* < .05). F, Left panel: Quantification of detectable CTC in patients before and after operation (*P* < .05). Right panel: Quantification of detectable CTC‐WBC clusters in patients before and after operation (*P* < .01). G, There was no correlation between IDH status and CTC levels in postoperative patients (*P* > .05). H, Left panel: higher level of CTC was observed in postoperative patients with P53 mutant (*P* < .05). Right panel: higher level of CTC‐WBC clusters was observed in postoperative patients with P53 mutant (*P* < .05)

**FIGURE 4:**
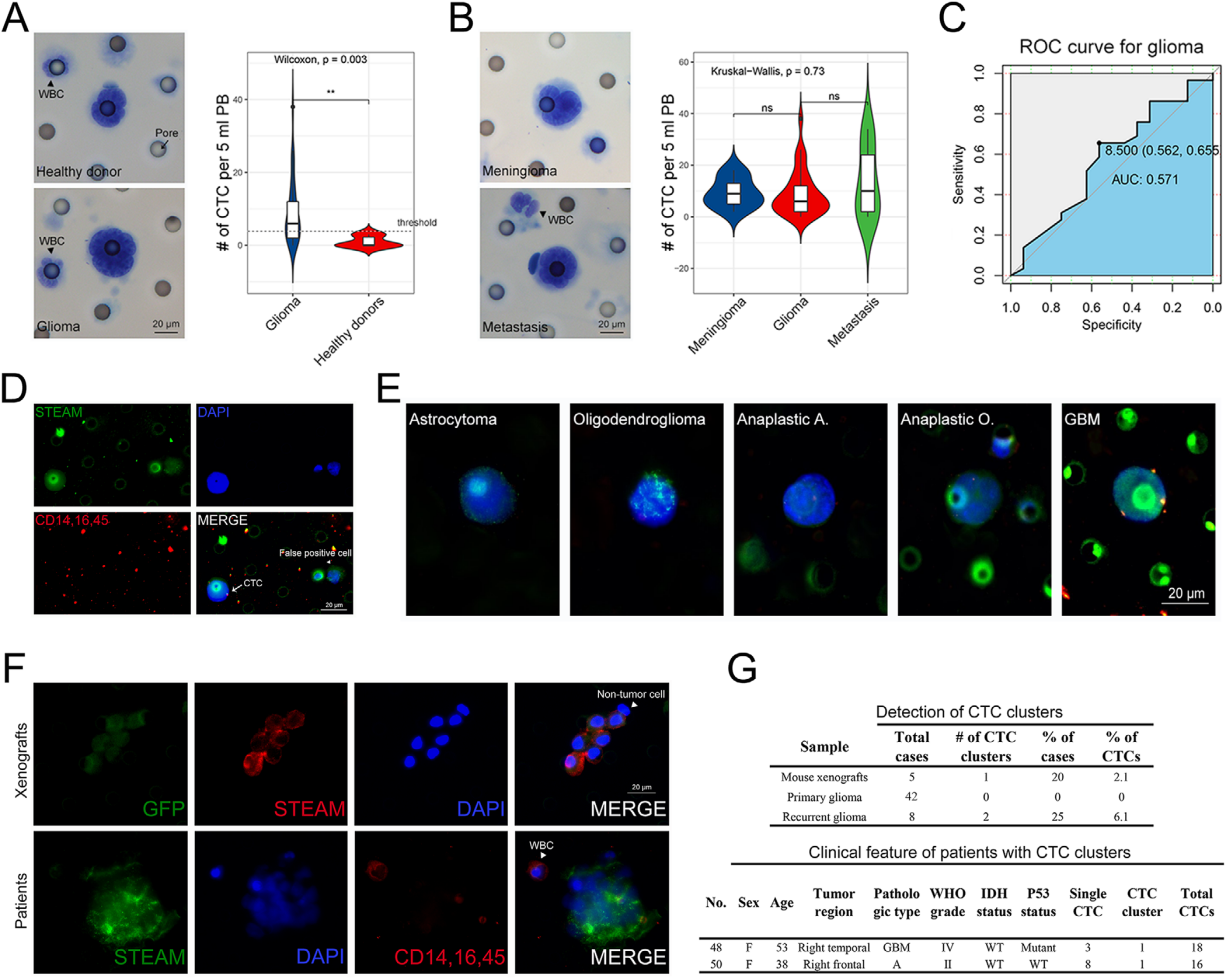
Supplemental information. A, Left panel: representative image of Wright's staining positive cells isolated from healthy donors (upper panel) and patients with glioma (lower panel). Scale bar = 20 μm. Right panel: quantification of Wright's staining positive CTCs in blood samples drawn from healthy donors (n = 6) and glioma patients (n = 29) (*P* < .01). Threshold: 4 Wright's staining positive cells per 10 ml PB. B, Left panel: representative image of Wright's staining positive cells isolated from patients with meningioma (upper panel) and metastasis (lower panel). Scale bar = 20 μm. Right panel: quantification of Wright's staining positive cells in blood samples drawn from patients with glioma (n = 29), meningioma (n = 9), and metastasis (n = 7) (*P* > .05). C, ROC curve analysis revealed that the level of Wright's staining positive CTC was not a good predictor of glioma (AUC = 0.571). D, Representative image for CTC and false positive cells by modified STEAM staining. Left cell: a STEAM‐positive CTC. Right: a false positive cell. Scale bar = 20 μm. The criteria for glioma CTC include: STEAM^+^/DAPI^+^/CD45^−^, nuclei larger than a 2‐calibrated pore size (8 μm) (ie. > 16 μm), irregular nuclei, and a high nuclear/cytoplasmic ratio. E, Representative image of five different pathological subtypes of glioma. Scale bar = 20 μm. F, Upper panel: representative image of CTC clusters isolated from mouse xenografts. Lower panel: representative image of CTC clusters isolated from glioma patients. Scale bar = 20 μm. G, Upper panel: quantification of detectable CTC clusters in xenografts (n = 5), primary glioma (n = 42), and recurrent glioma (n = 8). Lower panel: clinical feature of patients who had CTC clusters. A: astrocytoma; O: oligodendroglioma. GBM: glioblastoma multiforme

We then further investigate the correlation between CTC and patients’ clinical features. Our data revealed that CTC level was related to IDH status and poor outcomes (*P* < .05) (Figure 3E). Interestingly, we observed an impressive increase of CTC and clusters at 2 weeks after resection, indicating that resection might promote CTC entering circulation (Figure 3F). Moreover, the levels of postoperative CTC and CTC‐WBC clusters were positively related to P53 mutation (*P* < .05, respectively) rather than IDH status (Figure 3G). Above findings suggested that higher levels of CTC and clusters are not favorable predictors for glioma.

In conclusion, we present an efficient platform for detecting glioma CTC and their clusters with advantages of high sensitivity and specificity. We identified STEAM‐positive CTC as a highly specific diagnostic marker for glioma, which might be superior to MRI and biopsy to some extent. This finding is the strength of our study, highlighting the potential of liquid biopsy for clinical application. After validation in a larger cohort, CTC could be further used in pathological subtypes and molecular diagnosis.

Despite previous study highlighted the pro‐tumor effects of CTC‐WBC clusters,^10^ what role they have in glioma remain unclear. Thus related research are urgently required for understanding and evaluating their clinical value.

Our findings extremely expanded the clinical application of circulating glioma cell and their clusters, and also supply a bridge for CTC from basic research to clinical practice.

## ETHICS APPROVAL

The animal experiments of this research were approved by the Institutional Animal Care and Use Committee of the Renmin Hospital of Wuhan University. Clinical data were obtained from patients or healthy donors under Institutional Review Board‐approved protocols of the Renmin Hospital of Wuhan University.

## DATA AVAILABILITY STATEMENT

Data supporting the findings of this study are within this manuscript or available from the corresponding authors upon reasonable request.

## AUTHOR CONTRIBUTIONS

Y.Q, B.L, and Q.C designed this study. Y.Q, Q.S, G.D, Y.L, Y.L, Z.Y, and Y.W performed the experiments. Y.Q, H.Z, Y.X, S.H, F.Y, P.H, L.G, H.J, and W.W performed the clinical data collection and collation. All the authors were involved in the analysis and interpretation of data. Y.Q and G.D wrote the paper, with the help of the co‐authors. B.L and Q.C reviewed and revised the manuscript.

## CONFLICT OF INTEREST

The authors declare no potential conflicts of interest

## Supporting information

Fig S1

Fig S2

Fig S3

Fig S4

Supporting information

Table S1

Table S2

Table S3

Table S4

Table S5
