# Supplementary figures and images for: Identifying circulating glioma cells and their clusters as diagnostic markers by a novel detection platform
Source: Clin Transl Med. 2021 Feb 4;11(2):e318. doi: 10.1002/ctm2.318 (PMC7862585; doi:10.1002/ctm2.318)

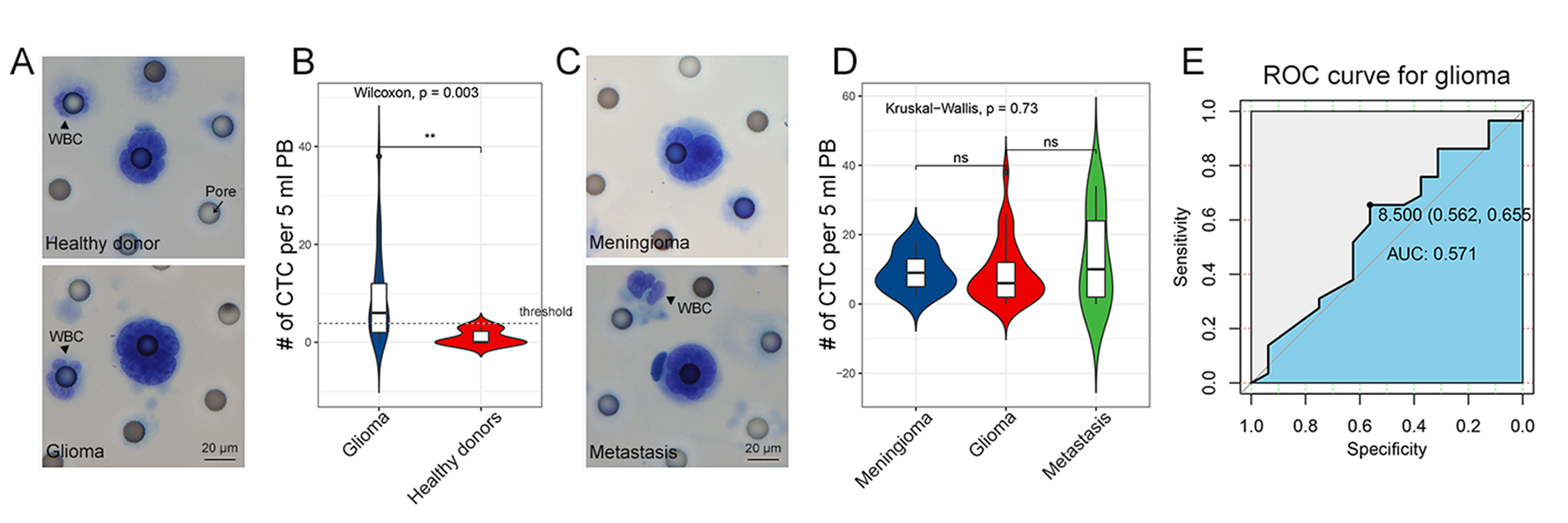

Supplement: Supplementary file 1 — Fig S1 [file CTM2-11-e318-s001.tif]

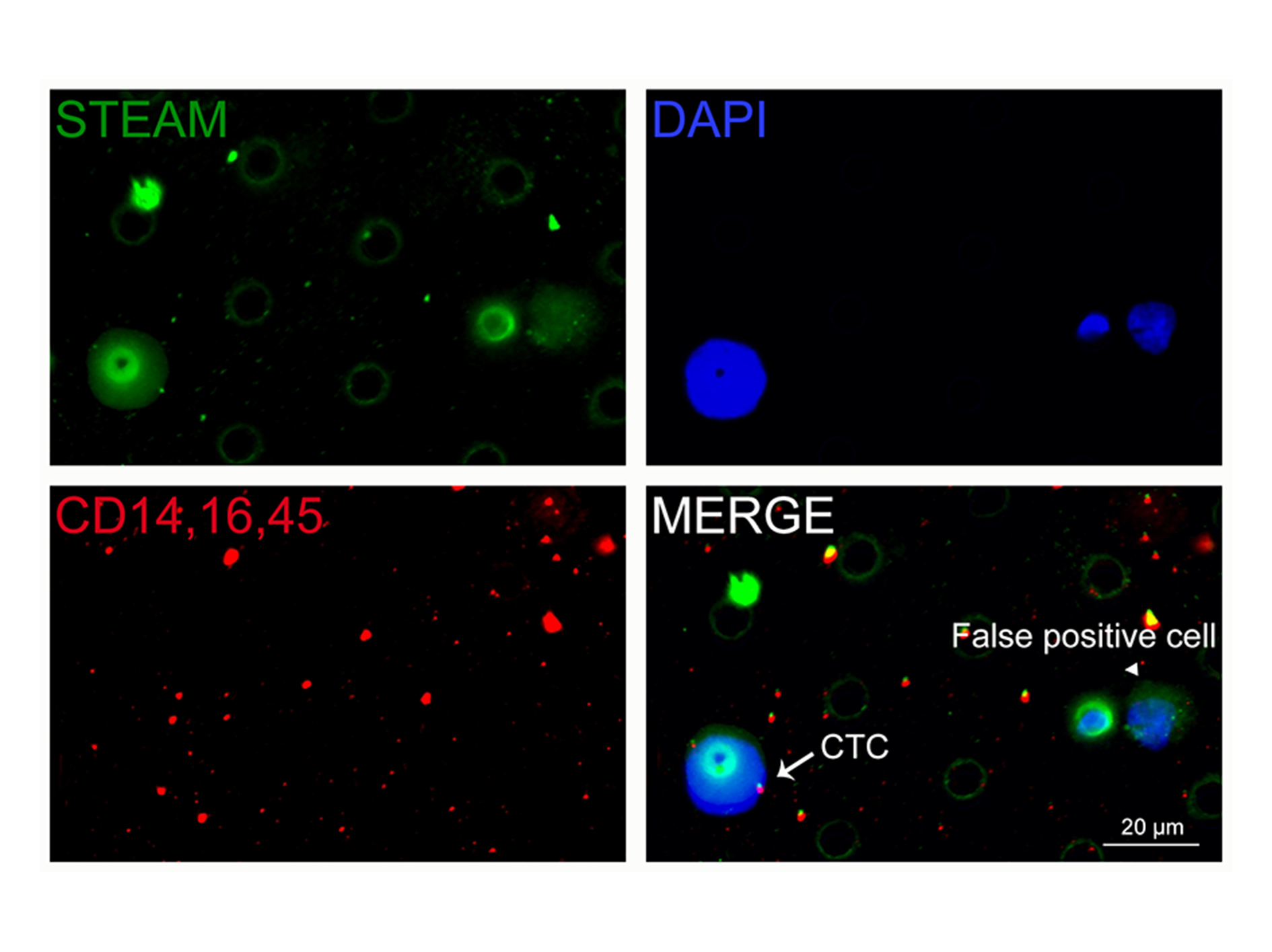

Supplement: Supplementary file 2 — Fig S2 [file CTM2-11-e318-s002.tif]

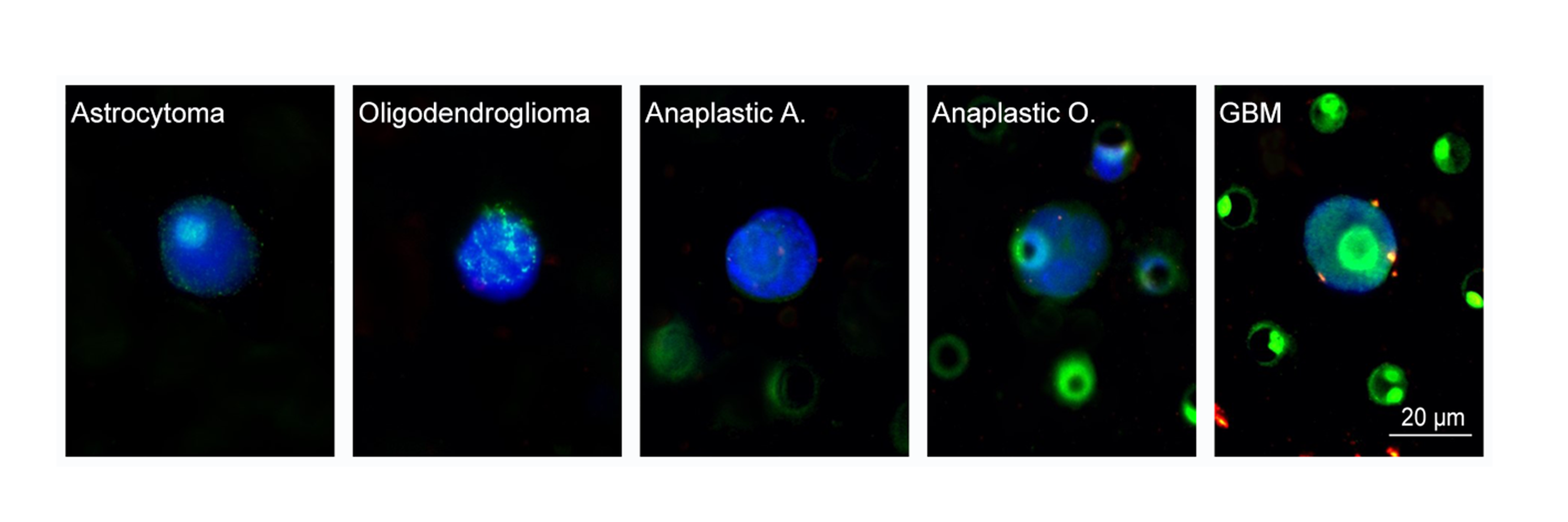

Supplement: Supplementary file 3 — Fig S3 [file CTM2-11-e318-s003.tif]

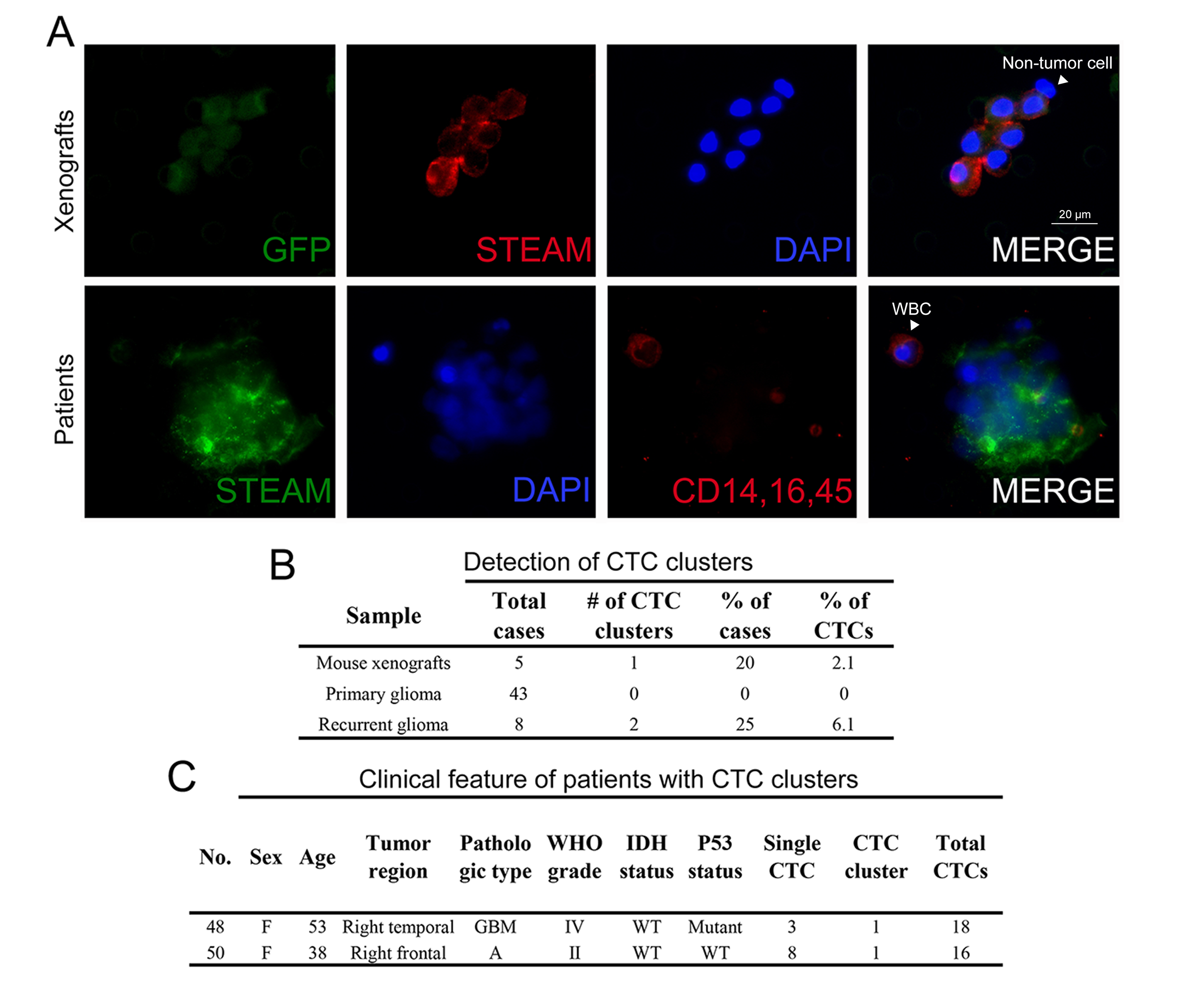

Supplement: Supplementary file 4 — Fig S4 [file CTM2-11-e318-s005.tif]
