# Supplementary material for: Identifying circulating glioma cells and their clusters as diagnostic markers by a novel detection platform
Source: Clin Transl Med. 2021 Feb 4;11(2):e318. doi: 10.1002/ctm2.318 (PMC7862585; doi:10.1002/ctm2.318)
Supplement: Supplementary file 5 — Supporting information [file CTM2-11-e318-s004.docx]

**Supplemental information**

**Methods and Materials**

***Cell Culture***

Human glioblastoma cell lines (U87 and U251) were purchased from the Cell Bank of the Shanghai Institute of Biochemistry and Cell Biology, Chinese Academy of Sciences (Shanghai, China). The STR authentications of U87 and U251 were performed by Procell Life Science & Technology Co., Ltd. (Wuhan, China) and are provided in the supplemental materials. All cell lines were cultured in Dulbecco’s modified Eagle’s medium (DMEM) (Genom, Hangzhou, China) with 10% fetal bovine serum (FBS) (Gibco, Thermo Fisher Scientific, USA) and 1% penicillin/streptomycin (Genom, Hangzhou, China). The cell incubation temperature was 37 ℃, with 5% CO_2_.

***Generation of Stable Cell Lines***

To establish the stable cell lines of U87-GFP and U251-GFP, lentiviral plasmid, pLVX-GFP-puro (Miaolingbio, Wuhan, China) was transfected with two helper vectors, pMD2.G (#12259, Addgene, USA) and psPAX2 (#12260, Addgene, USA) into 293T cells using Lipofectamine 2000 transfection reagent (Invitrogen, Carlsbad, CA, USA) to produce lentiviruses. The cells were infected with the lentiviruses and selected by puromycin (#73342, Stemcell, Canada) (5 mg/ml for U87 and 8 mg/ml for U251). The efficiency of viral infection was monitored by an Olympus BX51 microscope (Olympus, Tokyo, Japan).

***Isolation Procedure of the ISET Device***

In this device, the isolation is carried out using a biocompatible parylene polymer membrane with a pore diameter of 8 μ m under a high flow rate, enriching for CTCs without requiring tumor cell-specific capture antibodies. The ISET device is improved by applying automatic isolation without human intervention and could finish the whole procedure within 10 minutes, providing an advantage of high-throughput (Fig. 1A&B).

The blood sample (5 ml) was diluted 1:2 with BD wash buffer (BD, USA) containing 0.2% paraformaldehyde (PFA), 0.1% bovine serum albumin (BSA), and 0.0372% EDTA. It was incubated for 10 minutes at room temperature and then detected by the device. The filtrate was gently aspirated by a vacuum suction pump. After aspiration, the retained cells were washed three times with pure water and fixed in 100% methanol. After disassembly from the filter, the membrane was placed on a slide and coverslipped after it had air-dried.

***Capture Efficiency Test***

U87-GFP and U251-GFP cells were spiked into blood samples from healthy donors at a series of concentrations of 5, 10, 20, 50, 100, and 150 cells per 5 ml. The spiked samples were detected by the CTCBIOPSY® device. Each test was repeated three times. The identification of tumor cells was achieved by immunofluorescence and W&G staining.

***Wright’s Staining***

The slides were immersed in 100% xylene for several minutes at room temperature until the cover glasses dropped off. Then, we added eosin on the membrane for 2 minutes and then discarded it. Next, we added methylene blue for 1 minute, and then washed with PBS. Then, the membrane was air-dried and observed by light microscopy. The criteria for the identification of CTCs and CTC clusters used the cytomorphological criteria proposed by other research groups. The results of Wright’s staining were identified by two experienced cytopathologists.

***STEAM Staining***

STEAM staining was an antibody cocktail based on differential expressed genes between CTC and WBC, and had been proved to have ability to identifying GBM derived CTC from WBC in previous study.^3^ The antibody cocktail was annotated as STEAM by combining the initials of each selected genes (SOX2, Tubulin beta-3, EGFR, A2B5, and c-MET).

We fixed the captured cells on the membrane with 4% PFA for 5 minutes and subsequently washed it with PBS 3 times. Then, 150 μl 0.3% Triton-X 100 was added for 3 minutes in order to allow for intracellular staining. After that, we added 10% goat serum (Jackson ImmunoResearch) to block nonspecific binding for half an hour. Then, we discarded the serum and added the primary rabbit antibodies against Sox2 (CST), EGFR (CST), Met (CST), and Tubulin (Abcam), the mouse antibodies against CD14 (BD) and CD16 (Santa-Cruz), and the rat antibody against CD45 (Santa-Cruz) diluted 1:100 for incubation overnight at 4 ℃. On the next day, we washed the membrane with PBS 3 times and added the secondary Alexa Fluor 488 Goat anti-Rabbit IgG (Thermo Fisher), Alexa Fluor 546 Goat anti-Mouse IgG (Invitrogen), and Alexa Fluor 546 Goat anti-Rat IgG (Invitrogen) diluted 1:200 for incubation for 45 minutes at 37 ℃. The nuclei were stained with DAPI. The slides were imaged by an automated fluorescence microscopy scanning system (OLYMPUS IX81) under x40 magnification.

***Criteria for identification of glioma CTC***

The criteria for glioma CTC include: STEAM+/DAPI+/ED45-, nuclei larger than a 2-calibrated pore size (8 μm) (ie.>16 μm), irregular nuclei, and a high nuclear/cytoplasmic ratio.

***Generation, Raising and Treatment of Intracranial Xenograft Model***

Four-week-old BALB/c nude mice were purchased from Vital River Laboratory Animal Technology (Beijing, China) and kept in the Animal Experimental Center of Renmin Hospital of Wuhan University (Wuhan, China) for two weeks to adapt to the environment. According to the criterion of the ARRIVE checklist, our researchers checked the mice’s physical condition daily and chloral hydrate (350 mg/kg) was injected intraperitoneally for mice anesthesia during the operation. A total of 5×10^5^ cells in a 3 μL suspension of the U87-GFP stable cell line was injected at a depth of 3.5 mm from a skull hole that was drilled 3.5 mm from the cerebral midline and 2 mm frontal to the coronal suture, and a 3 μL injection of PBS was used as a control. Four weeks after the operation, all mice were sacrificed to collect CTCs from their peripheral blood. All mouse brains were ﬁxed in 4% paraformaldehyde and embedded in paraffin for further analysis. The animal experiments of this research were approved by the Institutional Animal Care and Use Committee of the Renmin Hospital of Wuhan University.

***Fluorescence in Vivo Imaging of Mouse Xenografts***

IR780 is a fluorescent dye employed for in vivo imaging. Betulinic acid (BA) nanoparticles (NPs) are a nano carrier synthesized in our laboratory [35]. IR780-loaded BA NPs were administered intravenously through the tail vein after the internal xenograft model mice were maintained for 4 weeks. The mice were imaged by a Bruker Xtreme BI imaging system with an excitation wavelength of 745 nm and an emission wavelength of 820 nm for free IR780 or IR780-loaded NPs 6 hours after injection. Mice were sacrificed to isolate the brain and other organs and imaged by the IVIS imaging system again. The fluorescence intensity in each brain was quantified using the Bruker MI SE 7.5 system.

***HE Staining***

HE staining of the sections was performed according to the routine protocols of hematoxylin-eosin staining. The images were captured with an Olympus BX51 microscope (Olympus, Tokyo, Japan).

***Blood Samples of Patients and Healthy Donors***

After receiving written informed consent, peripheral blood collections were obtained from patients or healthy donors under Institutional Review Board-approved protocols. All patients in the study were free of signiﬁcant comorbid medical conditions or prior cancer, deemed operable, and underwent a biopsy, subtotal, or gross total, surgical resection. Peripheral blood samples (5 ml × 2) were collected in EDTA buffer and processed by the device through the automatic isolation and staining procedure. All of the samples were collected before initial treatment and handled within 4 hours. For postoperative patients, peripheral blood samples (5 ml × 2) were collected 2 weeks after the operation.

***Statistical Analysis***

Statistical analysis was mainly performed with R (<https://www.r-project.org/>) with several publicly available packages. p<0.05 was considered to indicate statistical significance (^*^p<0.05, **p<0.01, ^***^p<0.001 and ^****^p<0.0001, respectively, as indicated in the figures and legends).
